# Rituximab for the treatment of immune checkpoint inhibitor–induced glomerulonephritis

**DOI:** 10.1093/ckj/sfaf373

**Published:** 2025-11-29

**Authors:** Yazen Alasadi, Juan Cintron Garcia, Naszrin Arani, Valda Page, Yimin Geng, Clark R Andersen, Amanda Tchakarov, Abhijat Kitchlu, Omar Mamlouk, Ala Abudayyeh

**Affiliations:** Section of Nephrology, Division of Internal Medicine, The University of Texas MD Anderson Cancer Center, Houston, TX, USA; Section of Nephrology, Division of Internal Medicine, The University of Texas MD Anderson Cancer Center, Houston, TX, USA; Section of Nephrology, Division of Internal Medicine, The University of Texas MD Anderson Cancer Center, Houston, TX, USA; Section of Nephrology, Division of Internal Medicine, The University of Texas MD Anderson Cancer Center, Houston, TX, USA; Research Medical Library, The University of Texas MD Anderson Cancer, Houston, TX, USA; Department of Biostatistics, The University of Texas MD Anderson Cancer Center, Houston, TX, USA; Department of Pathology and Laboratory Medicine, The University of Texas Health Science Center McGovern Medical School, Houston, TX, USA; Department of Medicine, Division of Nephrology, University Health Network, University of Toronto, Toronto, Ontario, Canada; Section of Nephrology, Division of Internal Medicine, The University of Texas MD Anderson Cancer Center, Houston, TX, USA; Section of Nephrology, Division of Internal Medicine, The University of Texas MD Anderson Cancer Center, Houston, TX, USA

**Keywords:** acute kidney injury, glomerulonephritis, immune checkpoint inhibitor, rituximab

## Abstract

**Background:**

Among patients receiving immune checkpoint inhibitor (ICI) therapy, the most common renal immune-related adverse event is interstitial nephritis, which is caused by severe T-cell infiltration and is typically responsive to steroids; although rarer, autoimmune induction in the kidney has also been reported. There is a paucity of data regarding ICI-induced autoimmune disease in the kidney and the challenges associated with continuing ICI therapy. Here, we present a single center case series of ICI-induced glomerulonephritis with data on treatment rechallenge.

**Methods:**

We retrospectively reviewed 241 patients who underwent kidney biopsies at our institution between 2015 and 2024 and identified those who had non-vasculitis-associated glomerulonephritis after ICI therapy. Demographics, renal toxicity, renal response, disease response, and overall survival data were extracted from the patients’ medical records. We defined renal response based on creatinine and proteinuria using KDIGO guidelines. We also performed a literature review to identify published cases of ICI-induced glomerulonephritis. Differences between treatment groups were assessed with a two-sided, two-sample *t*-test and Wilcoxon test or with a chi-square test, as appropriate.

**Results:**

Among 241 kidney biopsies we identified 16 patients with non-vasculitic GN, eight received rituximab with or without corticosteroids, and eight received corticosteroids only, for ICI-induced glomerulonephritis. The median proteinuria grades in the rituximab and corticosteroid groups were 3 and 1, respectively. The median corticosteroid treatment duration in the rituximab group (2.5 weeks) was shorter than that in the corticosteroid group (8 weeks). In the rituximab group, 75% of patients underwent ICI rechallenge and had no proteinuria relapse. Patients who received rituximab had better change in proteinuria response than those who received corticosteroids (*P* value = .029). Among the 42 patients we identified from the literature review, renal response did not differ significantly between the 10 who received rituximab and the 32 who did not.

**Conclusion:**

Use of Rituximab in treatment of ICI-induced GN is an attractive option to reduce steroids exposure allowing continued ICI treatment for overall improved renal outcome. Clinical trials to evaluate these findings are needed.

KEY LEARNING POINTS **What was known:**

- Interstitial nephritis is the most common renal immune-related adverse event in patients treated with immune checkpoint inhibitor (ICI).
- Although acute interstitial nephritis is the most commonly reported lesion on kidney biopsies, pathologies such as vasculitis and glomerular diseases have been reported. Other renal immune-related adverse events include: electrolyte disturbances, acute tubulointerstitial nephritis (ATIN), acute tubular necrosis, vasculitis, glomerulonephritis (GN), and, rarely, sarcoidosis.
- Guidelines for management of ICI-associated acute interstitial nephritis recommend glucocorticoid therapy.

**This study adds:**

- Glucocorticoid therapy in this setting raises concern for suppression of active T cells that target tumor cells, for which reduction of the dose and duration of steroids for ICI-associated acute interstitial nephritis is of key importance.
- Rituximab is an interesting option in the treatment of GN induced by ICI, considering reduction of glucocorticoid exposure.
- The use or Rituximab may also allow continued ICI treatment for overall improved renal outcome.

**Potential impact:**

- Kidney biopsies are necessary to both diagnose and tailor treatment for glomerulonephritis.
- We recommend the use of rituximab, instead of other cytotoxic therapies, to treat glomerulonephritis and non-crescentic vasculitis.
- Some patients with glomerulonephritis successfully treated with rituximab initially presenting with nephrotic range proteinuria had significant renal response and, in some cases, successfully rechallenged with ICI without renal relapse and continued cancer response.

## INTRODUCTION

In the last decade, multiple retrospective single and multicenter studies have evaluated the incidence of and risk factors for immune-related adverse events (irAEs) in the kidney [1]. Immune checkpoint inhibitor (ICI)-induced kidney injury occurs in 1.4%–16.5% of patients receiving ICI therapy [2]. The median time from ICI initiation to acute kidney injury (AKI) diagnosis ranges from 1 to 3 months [3–8], but AKI has also been reported as early as 3 days after ICI initiation and up to 72 weeks after ICI cessation [6, 9]. In previous cohorts of ICI-exposed patients, the incidence of AKI was 16.5%–17.0%, but only 3.0%–4.2% of AKI cases were attributed to ICI therapy [3, 5]. Renal irAEs vary; they include electrolyte disturbances, acute tubulointerstitial nephritis (ATIN), acute tubular necrosis, vasculitis, glomerulonephritis (GN), and, rarely, sarcoidosis. Although ATIN is the most commonly reported lesion on kidney biopsies other pathologies such as vasculitis and glomerular diseases has been reported [10, 11].

Current guidelines for managing new or relapsed ICI-associated ATIN recommend glucocorticoid-based therapy [12]. The increased use of ICIs (and therefore irAEs) has been accompanied by efforts to reduce the dose and duration of steroids for ICI-associated ATIN to avoid suppressing the active T cells that target tumor cells. Interest in steroid-sparing agents for renal irAEs has also grown, and proposed renal irAE treatment algorithms, extrapolated from findings from autoimmune disease treatment and tissue-based studies, include the use of cytokine-targeting agents and rituximab [13, 14]. Therefore, evaluating the use of rituximab as a first line therapy for ICI-induced GN that leads to renal dysfunction would help

understand its potential benefits in improving both renal and cancer outcomes.

## MATERIALS AND METHODS

### Patients

This retrospective study was approved by the Institutional Review Board at The University of Texas MD Anderson Cancer Center, and the procedures followed were in accordance with the principles of the Declaration of Helsinki. We identified patients listed in the MD Anderson pharmacy database who were treated with ipilimumab, nivolumab, pembrolizumab, atezolizumab, durvalumab, or avelumab between August 2015 and August 2024. The records of the 16 patients with glomerulonephritis were reviewed for baseline demographic characteristics; malignancy types; comorbidities; number of ICI cycles; concurrent use of nephrotoxic chemotherapy, proton pump inhibitors, and/or nonsteroidal anti-inflammatory drugs; urinalysis findings; proteinuria and other irAEs; cancer status at the time of AKI, at 3 and 6 months after AKI, and at last follow up; and survival data. Of note, patients were not involved in the design, conducting, reporting, or dissemination of plan in this study.

### Outcomes: renal response

Renal response to treatment was divided into creatinine response and proteinuria response. Creatinine response to treatment was evaluated at 3 months from time after the onset of AKI. Complete, partial, and stable creatinine responses were defined as a return of creatinine to baseline, any improvement in creatinine compared with baseline, and no change in creatinine compared with baseline, respectively. Proteinuria response was defined using the KDIGO guidelines: Complete, partial, and stable proteinuria responses (evaluated at the time of AKI) were defined as a spot urine protein-to-creatinine ratio ≤200 mg/g (or 0.2 g/24 h), a spot urine protein-to-creatinine ratio <2 g/g (or 2 g/24 h), and no change in proteinuria, respectively. Renal responses are included in supplemental Materials (S1).

### Outcomes: cancer response

Patients were evaluated at time of AKI at 3 months from GN treatment, at 6 months, and at last follow up. We assessed response by reviewing scans and oncology notes. Overall response was defined as having stable disease, remission, or any improved tumor response after treatment of the GN, and no response was defined as any progression of disease.

### Literature review

Since the incidence of ICI-induced GN has been reported to be <5%, we performed a literature review to further help understand the treatments at hand and outcomes. We used a search strategy we developed with a health information specialist (Y.G.) to search MEDLINE, the Cochrane Central Registry of Controlled Trials, and EMBASE for cases of ICI-associated glomerular disease published from February 2020 (the time of the last reported literature review) to February 2025 [15]. The search details and the terms used are included in the supplemental Materials (S2) [15]. We included all case reports, series, cohort studies, and clinical trials that reported a glomerular disease in association with the receipt of any ICI (including antibodies against CTLA4, PD1, or PDL1). We excluded studies that reported vasculitis or that did not have a documented kidney biopsy. Three nephrologists (J.C.G., O.M., and A.A.) screened all 816 articles and reviewed 44 articles in full. For each patient reported, we extracted relevant data, including demographic data, cancer diagnosis data, ICI treatment characteristics, laboratory data, glomerular disease-related descriptors, immunosuppressive treatment characteristics and AKI, proteinuria, and mortality outcomes.

### Statistical analyses

We analyzed the patients identified from the institutional database and those identified in the literature review separately. We summarized patients’ demographics and clinical characteristics overall and by rituximab treatment receipt. Differences between treatment groups were assessed with a two-sided, two-sample *t*-test and Wilcoxon test or with a chi-square test, as appropriate.

Utilizing the data from the literature, associations between renal response per creatinine and renal response per proteinuria were separately modeled by ordinal logistic regression with relation to rituximab treatment status, including creatinine at AKI as a covariate. Corresponding logistic regression models with Firth penalization were also produced which replaced ordinal response with binary response (PR or CR versus NR or SD) since these were considered more clinically relevant, although less powerful.

In all statistical testing, a 95% level of statistical confidence was assumed. All statistical analyses were performed using R statistical software version 4.4.1. Ordinal logistic regression used the “ordinal” package version 2023.12-4 [16]. Firth-penalized logistic regression used the “logistf” package version 1.26.0 [17].

## RESULTS

Among the patients we identified, 241 underwent a renal biopsy; of these, 105 had confirmed ATIN, including eight with thrombotic microangiopathy,16 with GN attributed to ICI, and 81 with vasculitis, hypertension, diabetes-related changes, or acute tubular necrosis. The median age of the 16 patients with glomerular disease after ICI therapy, was 67 years old, with 44% female and 56% males. The most common underlying cancer was genitourinary cancer (38%), followed by gastrointestinal cancer (19%) and lung cancer (19%). Three patients (19%) were treated with both anti-CTLA4 and anti-PDL1 ICIs. Renal pathologies were membranous nephropathy (31%), immunoglobulin A (IgA) nephropathy (25%), minimal change disease (19%), membranoproliferative glomerulonephritis (MPGN; 19%), and focal segmental glomerulosclerosis (FSGS; 6%). Proteinuria ranged from 0.8 to 20 g

Five patients (31%) had other irAEs such as hypophysitis, pancreatitis, and hypothyroidism. Two patients also received nonsteroidal anti-inflammatory drugs during ICI therapy. Three patients had prior autoimmune disease; one had hypothyroidism, and the other two had membranous nephropathy >10 years before initiating ICI therapy. Six of the 16 patients were alive at last follow up.

Six of the 16 patients-initiated steroids prior to biopsy. Of the eight patients treated with rituximab, only two received plasmapheresis. Patients treated with rituximab were given two doses of 1 g IV rituximab 2 weeks apart.

The rest of the patients were treated with steroids. The median time from start of ICI to AKI and associated proteinuria was 126 days. The median number of ICI cycles was 4, and the median time from ICI initiation to AKI and associated proteinuria was 104 days. For all patients, the median steroid exposure duration was 3.5 weeks (range, 0–112 weeks). The median steroid exposure duration for patients treated with rituximab (2.5 weeks; range, 0–27 weeks) was shorter than that for patients treated with steroids only (8 weeks; range, 1–112 weeks).

### Renal response

Among the eight patients treated with steroids, four had complete creatinine response and four had partial creatinine response. In addition, five had complete proteinuria response, two had stable proteinuria response, and one had no proteinuria response; however, five had <1 g of proteinuria at the time of AKI. Among the eight patients treated with rituximab, seven had complete creatinine response and one had partial creatinine response; two had complete proteinuria response, five had partial proteinuria response, and one had no proteinuria response. Compared with those who did not receive rituximab, patients who received rituximab had a higher median proteinuria 10.8 g (range, 3.15–20 g) vs 0.5 g (range 0–9.69) and higher interstitial fibrosis, tubular atrophy, and glomerulosclerosis (median glomerulosclerosis rituximab treated 23% vs 10% in non-rituximab treated). Among the patients with proteinuria of >1 g at baseline, the patients treated with rituximab had >50% to 97% decline in proteinuria and 83% decline in proteinuria in one patient treated with prednisone for 4 weeks. Among the patients treated with rituximab, minimal change disease and membranous nephropathy were the more common pathologies with no significant difference in creatinine or proteinuria response in either group. Only three patients with membranous nephropathy were tested for anti-PLA2R with one out of three was positive. Two of the 16 patients needed dialysis at the time of AKI and were diagnosed with MPGN, which was treated with steroids only. One patient had complete creatinine and proteinuria responses, and the other patient had a partial creatinine response but no proteinuria response (Table 1).

**Table 1:** Characteristics of MD Anderson patients by rituximab treatment status.

Characteristic	Patients without rituximab treatment (*N* = 8)	Patients with rituximab treatment (*N* = 8)	All patients (*N* = 16)	*P* value	Wilcoxon *P* value
Age, years					
Mean (SD)	61.7 (15.7)	63.8 (8.84)	62.7 (12.4)	.75	.80
Median (range)	66.0 (31.3–78.3)	67.0 (45.5–71.4)	67.0 (31.3–78.3)		
Sex, *n* (%)					
Female	5 (63)	2 (25)	7 (44)	.31	
Male	3 (38)	6 (75)	9 (56)		
Creatinine at AKI, mg/dl					
Mean (SD)	2.81 (1.95)	2.07 (1.05)	2.44 (1.56)	.36	.64
Median (range)	2.30 (0.910–7.04)	1.92 (0.800–3.87)	2.30 (0.800–7.04)		
Proteinuria at diagnosis, g					
Mean (SD)	2.03 (3.37)	11.2 (5.38)	6.61 (6.41)	.002	.004
Median (range)	0.550 (0–9.69)	10.8 (3.15–20.0)	5.53 (0–20.0)		
Proteinuria grade					
Mean (SD)	1.50 (1.07)	2.88 (0.354)	2.19 (1.05)	.008	.010
Median (range)	1.00 (0–3.00)	3.00 (2.00–3.00)	3.00 (0–3.00)		
Dialysis, *n* (%)					
No	6 (75)	8 (100)	14 (88)	.47	
Yes	2 (25)	0 (0)	2 (13)		
Biopsy specimen pathology, *n* (%)					
FSGS	0 (0)	1 (13)	1 (6)	.09	
IgA nephropathy	3 (38)	1 (13)	4 (25)		
MCD	0 (0)	3 (38)	3 (19)		
MN	2 (25)	3 (38)	5 (31)		
MPGN	3 (38)	0 (0)	3 (19)		
Steroids, *n* (%)					
No	0 (0)	1 (13)	1 (6)	1	
Yes	8 (100)	7 (88)	15 (94)		
Cancer type, *n* (%)					
Gastrointestinal	2 (25)	1 (13)	3 (19)	.66	
Genitourinary	3 (38)	3 (38)	6 (38)		
Lung	2 (25)	1 (13)	3 (19)		
Lymphoma	1 (13)	0 (0)	1 (6)		
Head and neck	0 (0)	1 (13)	1 (6)		
Melanoma	0 (0)	2 (25)	2 (13)		
ICI type, *n* (%)					
Anti-CTLA4	0 (0)	0 (0)	0 (0)	1	
Anti-PDL1	8 (100)	8 (100)	16 (100)		
Combination ICI, *n* (%)					
No	7 (88)	6 (75)	13 (81)	1	
Yes	1 (13)	2 (25)	3 (19)		
ICI rechallenge, *n* (%)					
No	6 (75)	2 (25)	8 (50)	.13	
Yes	2 (25)	6 (75)	8 (50)		
Overall response: creatinine, *n* (%)					
Yes	8 (100)	8 (100)	16 (100)		
No	0 (0)	0 (0)	0 (0)		
Overall response: proteinuria, *n* (%)					
Yes	5 (63)	7 (88)	12 (75)	.57	
No	3 (38)	1 (13)	4 (25)		

### ICI rechallenge

**Figure 1:**
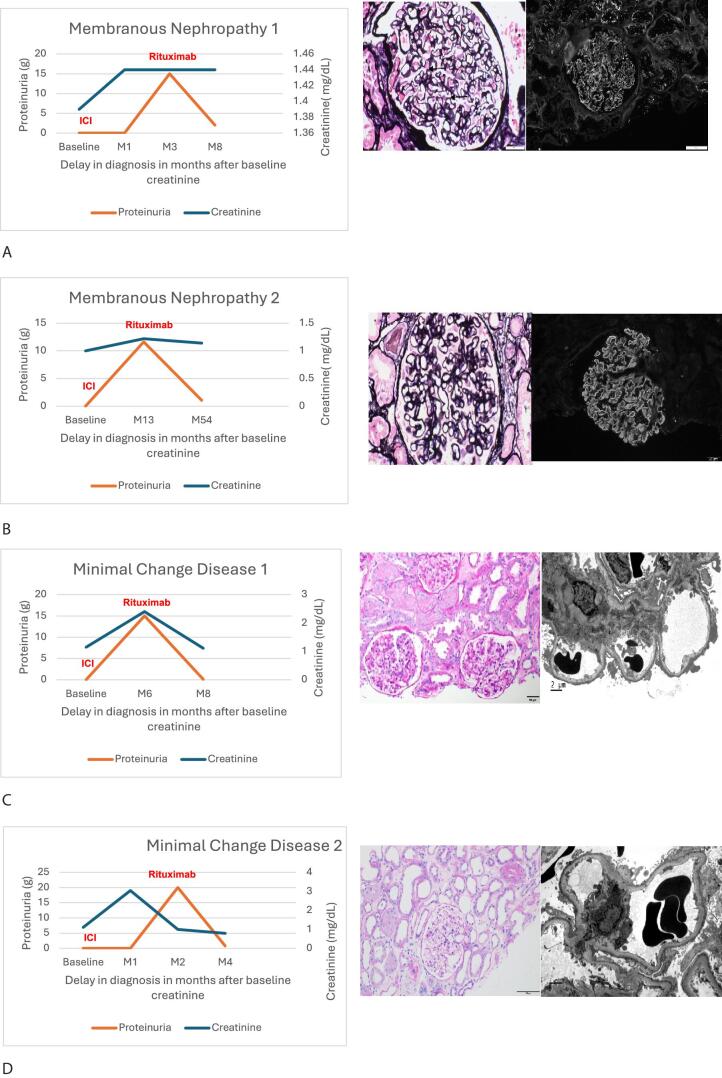
Cases of patients who underwent ICI rechallenge after rituximab treatment. (**A**) Patient in their 60s who had primary membranous nephropathy 20 years ago experienced reactivation of the nephropathy after receiving ICI therapy for stage IV melanoma. The reactivated nephropathy was exacerbated by ICI therapy. One cycle of rituximab achieved excellent response and proteinuria resolution, which were maintained for more than two additional years of ICI therapy without exacerbation of the membranous nephropathy. (**B**) Patient in their 60s with mesothelioma developed nephrotic range proteinuria after two cycles of nivolumab. Biopsy specimen analysis confirmed membranous nephropathy with anti-PLA2R antibodies. The patient-initiated rituximab and had renal recovery; continued ICI therapy with response for 2 years. (**C**) Patient in their 70s with anal canal squamous cell carcinoma that did not respond to fluorouracil was started on nivolumab and developed nephrotic range proteinuria after the fourth cycle. Biopsy specimen analysis confirmed minimal change disease. The patient was treated with a course of steroids and rituximab. Had complete remission of the proteinuria and continued ICI therapy with cancer response. (**D**) Patient in their 60s with anaplastic thyroid carcinoma received dabrafenib, trametinib, and pembrolizumab. Two weeks after initiating pembrolizumab, the patient developed AKI with an increased creatinine level of 3.5 mg/dl and nephrotic range proteinuria. Started on steroids, and biopsy specimen analysis confirmed minimal change disease. The patient was treated with rituximab, and proteinuria improved from 20 to 0.5 g. The patient was restarted on pembrolizumab with continued renal response but tumor progression.

Of the 16 patients, eight underwent ICI rechallenge. Six patients received rituximab for glomerulonephritis prior to ICI rechallenge and did not have glomerulonephritis relapse after ICI rechallenge (three MCD and three MN) ; some of these patients’ cases are illustrated in Fig. 1. Only two patients who received only steroids for glomerulonephritis (IgAN) underwent ICI rechallenge; however, they had milder proteinuria (≤1 g) at the time of AKI.

### Cancer response

Among the eight patients treated with rituximab, three had a partial or complete tumor response and five had progressive disease at last follow up. Similarly, among the eight patients treated with steroids, three had a partial or complete tumor response and five had progressive disease at last follow up. The median overall survival duration of the patients treated with rituximab (499 days) was similar to that of the patients treated with steroids (498 days). Among the patients who were rechallenged with ICI, seven out of eight had cancer response at 3 months, four out of eight at 6 months, and three out of eight had cancer response at last follow up.

### Literature review results

From the 44 articles that were selected for final review, we identified 51 patients with ICI-induced glomerulonephritis (Table S2). Excluding patients whose renal outcomes and treatment interventions were unavailable yielded a final list of 42 patients who were evaluated for renal outcomes based on creatinine or proteinuria response. Among these patients, the most common renal pathology was membranous nephropathy (38%), followed by IgA nephropathy (33%), minimal change disease (19%), FSGS (7%), and MPGN (2%). The most common cancer associated with ICI-induced GN was lung cancer (45%) followed by genitourinary cancer (17%) and melanoma (14%). The median proteinuria at diagnosis was 7.7 g (range, 0.3–20.2 g), and the median creatinine at AKI was 2.30 mg/dl (range, 0.790–9.40 mg/dl). Ten percent of the patients underwent dialysis. Of the 42 patients, 10 (24%) were treated with rituximab, of whom eight (80%) were diagnosed with membranous nephropathy. Only 10% of the patients underwent ICI rechallenge; half had received rituximab before rechallenge. The overall creatinine response was 84%, and the overall proteinuria response was 83%. The creatinine and proteinuria response rates of the patients who received rituximab (both 100%) were numerically higher than those of the patients who did not receive rituximab (79% and 78%, respectively), but these differences were not statistically significant (*P* = .17 and *P* = .30, respectively) (Table 2). Of the cases reviewed in the literature and reported in Table 2, four of patients who were treated with rituximab had missing creatinine or proteinuria data.

**Table 2:** Characteristics of literature review: identified patients by rituximab treatment status.

Characteristic	Patients without rituximab treatment (*N* = 32)	Patients with rituximab treatment (*N* = 10)	All patients (*N* = 42)	*P* value	Wilcoxon *P* value
Age, years					
Mean (SD)	63.4 (16.7)	68.6 (8.30)	64.7 (15.2)	.20	.49
Median (range)	69.5 (7.00–88.0)	71.5 (56.0–80.0)	70.5 (7.00–88.0)		
Sex, *n* (%)					
Female	7 (22)	0 (0)	7 (17)	.17	
Male	25 (78)	10 (100)	35 (83)		
Creatinine at AKI, mg/dl					
Mean (SD)	2.97 (1.90)	2.34 (1.73)	2.83 (1.86)	.43	.25
Median (range)	2.40 (0.790–9.40)	2.21 (0.840–6.00)	2.30 (0.790–9.40)		
Proteinuria at diagnosis, g					
Mean (SD)	9.28 (6.47)	7.85 (6.31)	8.87 (6.36)	.55	.73
Median (range)	9.90 (0.300–19.9)	5.45 (0.420–20.2)	7.70 (0.3–20.2)		
Proteinuria grade					
Mean (SD)	2.72 (0.542)	2.70 (0.675)	2.71 (0.572)	.93	.90
Median (range)	3.00 (1.00–3.00)	3.00 (1.00–3.00)	3.00 (1.00–3.00)		
Dialysis, *n* (%)					
No	29 (91)	8 (80)	37 (88)	.25	
Yes	2 (6)	2 (20)	4 (10)		
Biopsy specimen pathology, *n* (%)					
FSGS	3 (9)	0 (0)	3 (7)	.026	
IgA nephropathy	13 (41)	1 (10)	14 (33)		
MCD	7 (22)	1 (10)	8 (19)		
MN	8 (25)	8 (80)	16 (38)		
MPGN	1 (3)	0 (0)	1 (2)		
Steroids, *n* (%)					
No	5 (16)	4 (40)	9 (21)	.18	
Yes	27 (84)	6 (60)	33 (79)		
Cancer type, *n* (%)					
Gastrointestinal	2 (6)	1 (10)	3 (7)	.61	
Genitourinary	4 (13)	3 (30)	7 (17)		
Head and neck	3 (9)	0 (0)	3 (7)		
Lung	14 (44)	5 (50)	19 (45)		
Lymphoma	4 (13)	0 (0)	4 (10)		
Melanoma	5 (16)	1 (10)	6 (14)		
ICI type, *n* (%)					
Anti-CTLA4	2 (6)	0 (0)	2 (5)	1	
Anti-PDL1	30 (94)	10 (100)	40 (95)		
Combination ICI, *n* (%)					
No	28 (88)	8 (80)	36 (86)	.62	
Yes	4 (13)	2 (20)	6 (14)		
ICI rechallenge, *n* (%)					
No	30 (94)	8 (80)	38 (91)	.24	
Yes	2 (6)	2 (20)	4 (10)		
Any response: creatinine, *n* (%)					
Yes	22 (79)	10 (100)	32 (84)	.17	
No	6 (21)	0 (0)	6 (16)		
Any response: proteinuria, *n* (%)					
Yes	21 (78)	8 (100)	29 (83)	.30	
No	6 (22)	0 (0)	6 (17)		

### Statistical modeling

Statistical modeling focused on the data from the literature since the local institutional data indicated confounding between rituximab treatment and proteinuria (see Table 1); models including both datasets as well as local institutional data alone were explored but did not yield different conclusions and are omitted to focus on the relatively unbiased literature data. Inclusion of other baseline variables was explored, but all led to a worse model based on the Akaike information criterion.

The ordinal logistic regression model of renal response per creatinine (with ordered responses CR > PR > NR; none had SD) showed no significant evidence of association with rituximab treatment status (an odds ratio of 3.04 with 95% confidence interval spanning 0.54–17.04, *P* = .21), though it did show evidence of improved response with higher creatinine at AKI (odds ratio of 0.61 per unit increase of creatinine, with 95% confidence interval spanning 0.38–0.96, *P* = .032; note that for ordinal logistic regression the odds ratio refers to the odds of a higher level response versus a lower level response). The corresponding logistic regression model of any renal response per creatinine (with binary response defined as PR or CR versus NR) showed no evidence of association with rituximab treatment status [OR 4.02, 95% confidence interval (CI) 0.34–573.4, *P* = .31], nor was there significant evidence of association with creatinine at AKI (OR 0.66, 95% CI 0.33–1.02, *P* = .062).

The ordinal logistic regression model of renal response per proteinuria (with ordered responses CR > PR > SD > NR) showed no significant evidence of association with rituximab treatment status [an odds ratio (OR) of 2.21 with 95% CI spanning 0.32–15.36, *P* = .42], although it did show evidence of improved response with higher creatinine at AKI (OR 0.54 per unit increase of creatinine, with 95% CI 0.30–0.98, *P* = .043). The corresponding logistic regression model of any renal response per proteinuria (with binary response of PR or CR versus NR or SD) showed no evidence of association with rituximab treatment status (OR 3.36, 95% CI 0.26–478.56, *P* = .39), nor was there significant evidence of association with creatinine at AKI (OR 0.66, 95% CI 0.28–1.03, *P* = .07).

## DISCUSSION

ICI-induced glomerulonephritis is rare but can complicate patient care and negatively affect survival. In a recent meta-analysis, the most common ICI-associated glomerular disease was pauci-immune glomerulonephritis/renal vasculitis (27%), followed by minimal change disease (20%) and C3GN (11%); 41% of the patients had concomitant ATIN; however, no clear conclusions could be drawn about use of rituximab as a treatment of ICI-induced GN [15]. The median time to glomerular disease diagnosis after ICI initiation was 93 days (interquartile range, 44–212 days). Most patients (98%) received corticosteroids, and these patients had rates of complete and partial recovery from AKI of 31% and 42%, respectively. Approximately 19% of the patients underwent dialysis, and of these patients, approximately one-third died.

In the present study of a limited number of cases of ICI-induced glomerulonephritis, we sought to evaluate renal outcomes with currently available treatments and interventions. We found no statistically significant difference in between patients treated with rituximab and those treated with steroids, but all patients treated with rituximab had renal responses, as evidenced by improvements in both serum creatinine and proteinuria. In addition, patients treated with rituximab were more likely to undergo ICI rechallenge and had less exposure to steroids. However, ordinal and binary logistic regression models found no evidence of association between rituximab treatment and renal response after controlling for creatinine at AKI.

Kidney biopsies are necessary to both diagnose and tailor treatment for glomerulonephritis. This recommendation is a departure from some current guidelines for the management of renal irAEs, which suggest forgoing kidney biopsy and immediately starting corticosteroid-based treatment [18]. Although patients who develop glomerulonephritis may have to stop ICI therapy, several case reports have demonstrated that treatment with rituximab enabled patients to continue ICI therapy and have both renal recovery and disease remission [15, 19].

ICI therapy may induce autoimmune disease in the kidney by suppressing regulatory T cells, thereby increasing the risk of antibody-mediated autoimmune diseases and upregulating the cytokines interferon-γ and interleukin-12. These cytokines’ upregulation has been associated with the increased interaction of B lymphocytes with expanded T-lymphocyte populations and the development of anti-neutrophil cytoplasmic antibody vasculitis [20–24]. In addition, ICIs have been reported to upregulate CXCL9 and CXCL10, which facilitate T-cell recruitment and have been associated with tissue injury in patients with IgA vasculitis [25, 26].

In our experience, we recommend using rituximab, as opposed to other cytotoxic therapies, to treat glomerulonephritis and non-crescentic vasculitis. Rituximab, a monoclonal anti-CD20 antibody that disrupts pathogenic B lymphocytes’ interaction with cytotoxic T lymphocytes, reduces chemokine production, and limits endothelial injury, has not yet been shown to inhibit the anti-neoplastic effects of ICI therapy [27, 28]. In a small cohort of five patients diagnosed with ICI-induced renal vasculitis, treatment with rituximab resulted in partial to complete renal recovery and no renal relapses [29].

Our study had several limitations that hindered a valid comparison between rituximab treated and non-rituximab treated ICI-induced GN. For example, among patients in the MD Anderson cohort, those who were treated with rituximab had a higher median proteinuria than those who were not treated with rituximab (3 g vs ≤1 g). In addition, rituximab treated group were more likely FSGS, minimal change disease, or membranous nephropathy compared to the non-rituximab group, which would be consistent with higher levels of podocytopathy and therefore rituximab would result in a greater reduction in proteinuria. Due to retrospective nature of our study, the patients were not tested for CD19 after rituximab treatment, anti-rituximab testing, or relevant autoantibodies to correlate with response before and after treatment. The patients identified from the literature review had missing data, including actual proteinuria values, as well as limited exposure to rituximab, which limited the strength of the analysis. These limitations prevented a more robust statistical evaluation of the impact of rituximab on renal response after ICI-induced GN and overall survival.

## CONCLUSION

ICI-induced glomerular disease is rare but presents a challenge in the treatment of cancer patients and the continuation of ICI therapy. Here, we present patients with glomerulonephritis who were successfully treated with rituximab with nephrotic range proteinuria and significant renal response and in some cases successful rechallenge with ICI without renal relapse and continued cancer response. A definitive, multicenter study of ICI-induced glomerulonephritis, its impact on both renal and tumor response, and its effective treatments is warranted.

## Supplementary Material

sfaf373_Supplemental_File

## Data Availability

All data generated or analyzed during this study are included in this published article.
