# Supplementary material for: Rituximab for the treatment of immune checkpoint inhibitor–induced glomerulonephritis
Source: Clin Kidney J. 2025 Nov 29;19(1):sfaf373. doi: 10.1093/ckj/sfaf373 (PMC12957936; doi:10.1093/ckj/sfaf373)
Supplement: sfaf373_Supplemental_File [file sfaf373_Supplemental_File.pdf]

Supplementary:

Supplementary Material – Literature Search Strategy

Date: February 4, 2025

Information Specialist:

Yimin Geng, MS, MSLIS

Library Information Analyst Research Medical Library

The University of Texas MD Anderson Cancer

713-563-8018

ORCID: orcid.org/0000-0002-8491-3151

Email: ygeng@mdanderson.org

Research Question

[What literature is available on the immune-related adverse effects of immune checkpoint inhibitors on the glomerulus?]

Description

The search strategy takes the format of Immune Checkpoint Inhibitors AND Glomerular Disease.

Limits

The search has been limited to English language articles, published from Feb 2020 to the Feb 2025 . Animal-only studies have also been removed.

Number of results per database

Databases Searched Date of Search Number of Results

All Ovid Medline

EBM Reviews - Cochrane Central Register of Controlled Trials

2/4/2025

Database(s): Embase Classic+Embase 1947 to 2025 February 03

Search Strategy:

#	Searches	Results
1	immunotherapy/ae [Adverse Drug Reaction]	148
2	ipilimumab/	28355
3	pembrolizumab/	46073
4	atezolizumab/	20039
5	avelumab/	7666
6	durvalumab/	13247
7	nivolumab/	45266
8	cemiplimab/	2538
9	(check?point adj2 inhibit*).ti,ab.	63995

10	(check?point adj2 block*).ti,ab.	18007
11	CTLA-4 blockade*.ti,ab.	1072
12	Ipilimumab.ti,ab.	12285
13	Yervoy.ti,ab.	127
14	PD-1 inhibitor*.ti,ab.	6361
15	Anti-PD-1.ti,ab.	20683
16	Pembrolizumab.ti,ab.	23157
17	Keytruda.ti,ab.	364
18	PD-L1 inhibitor*.ti,ab.	4604
19	Anti-PD-L1.ti,ab.	7794
20	Atezolizumab.ti,ab.	8290
21	Tecentriq.ti,ab.	85
22	Avelumab.ti,ab.	2467
23	Bavencio.ti,ab.	19

24	Durvalumab.ti,ab.	5090
25	Imfinzi.ti,ab.	37
26	Nivolumab.ti,ab.	23145
27	Opdivo.ti,ab.	179
28	Cemiplimab.ti,ab.	1066
29	Libtayo.ti,ab.	22
30	or/1-29	151437
31	*acute kidney failure/	54758
32	*acute kidney tubule necrosis/	1206
33	exp *Nephritis/	89390
34	*AA Amyloidosis/	546
35	exp *Nephrosis/	35834
36	*Immunoglobulin A nephropathy/	10035
37	exp *Glomerulopathy/	57881

38	(acute kidney adj5 (injur* or insufficienc* or failure*)).ti.	30659
39	AKI.ti.	5842
40	(acute renal adj5 (injur* or insufficienc* or failure*)).ti.	15056
41	nephritis.ti. or nephritis.ab. /freq=2	27989
42	glomerul*.ti. or glomerul*.ab. /freq=2	106952
43	amyloidos?s.ti.	28004
44	nephropath*.ti.	49078
45	minimal change disease*.ti.	863
46	acute tubular necrosis.ti.	802
47	nephrotoxicit*.ti.	10126
48	kidney toxicit*.ti.	162
49	nephros?s.ti.	3190
50	or/31-49	325056
51	30 and 50	716

52	limit 51 to english language	701
53	(Nonhuman/ or ANIMAL/ or Animal Experiment/) not Human/	7506655
54	52 not 53	682
55	(mice or mouse or murine or rat or rats or rodent or cells or "in vitro" or "cell line").ti.	3617128
56	54 not 55	671
57	limit 56 to dc="20100101 -20200131" [from 2010 to January 2020]	106
58	limit 56 to dc=" 20200201-20250129" [February 2020 to current]	563
59	conference abstract.pt.	5356225
60	58 and 59	265

"/" indicates "subject heading"

Supplementary:

Table S1: Summary of patient review data

Reference	Age	Sex	Cancer Site	ICI	Baseline Creatinine (mg/dL)	Peak Creatinine (mg/dL)	Peak Proteinuria (Random spot protein or 24hr: g)	Renal Lesion	Corticosteroid Therapy	Other Treatment Given	Post Treatment Proteinuria (Random spot protein or 24hr: g g)	Post Treatment Creatinine (mg/dL)	Outcome Creatinine	Outcome Proteinuria	Cancer Status
1	70	Female	Ovarian	Pembrolizumab	1.07	1.35	7.00	FSGS	No Steroids	Rituximab	3.40	1.12	CR	PR	No Response
2	45	Male	RCC	Pembrolizumab	0.6	0.8	10.00	MCD	Steroids	Rituximab	1.82	0.67	CR	PR	No Response
3	71	Male	RCC	Nivolumab	1.41	2.41	1.14	IgA GN	Steroids	No Rituximab	1.27	1.48	CR	SD	No Response
4	68	Male	Melanoma	Ipilumamab and Nivolumab	1.39	1.44	15.00	MN	No Steroids	Rituximab	1.98	1.44	CR	SD	Response
5	54	Male	GI	Pembrolizumab	1.43	2.28	0.50	MPGN	Steroids	No Rituximab	0.20	1.58	CR	CR	No Response
6	61	Male	Lung	Nivolumab	1.00	1.22	11.63	MN	No Steroids	Rituximab	1.06	1.14	CR	PR	Response
7	78	Female	GI	Atezolumab	0.77	7.04	0.25	MPGN	Steroids	No Rituximab	0.40	1.02	PR	SD	No Response
8	71	Female	GI	Nivolumab	1.15	2.4	15.00	MCD	Steroids	Rituximab	0.13	1.11	CR	CR	No Response
9	74	Female	RCC	Nivolumab	1.41	2.13	0.60	MN	Steroids	No Rituximab	0.10	1.70	PR	CR	No Response
10	57	Male	RCC	Pembrolizumab	1.05	3.87	3.15	MN	Steroids	Rituximab	1.20	1.50	PR	PR	No Response
11	31	Male	Myeloid Sarcoma	Ipilumamab and Nivolumab	0.86	2.32	0.03	IgA GN	Steroids	No Rituximab	0.00	0.79	CR	CR	No Response
12	65	Male	Thyroid	Pembrolizumab	1.11	3.04	20.00	MCD	Steroids	Rituximab	0.82	1.00	CR	CR	No Response
13	50	Female	Lung	Pembrolizumab	0.5	1.27	0.00	IgA GN	Steroids	No Rituximab	0.00	1.68	PR	CR	Response
14	61	Female	RCC	Nivolumab	0.67	0.91	9.69	MN	Steroids	No Rituximab	0.20	0.57	CR	CR	Response
15	69	Male	Melanoma	Ipilumamab and Nivolumab	1.44	2.4	7.70	IgA GN	Steroids, Acthar	Rituximab	1.32	1.30	CR	PR	Response
16	72	Female	Lung Cancer	Nivolumab	0.94	4.11	4.06	MPGN	Steroids	No Rituximab	UA 10 from UA >500, 3 consecutive occasions	2.19	PR	NR	Response

Table S2: Summary of literature review data

Reference	Age	Sex	Cancer site	ICI	Baseline Cr	Peak Cr	Peak proteinuria (Random spot protein or 24hr: g, or UPC ratio (g/g))	Renal lesion	Corticosteroid therapy	Other treatment given	Post treatment proteinuria (Random spot protein or 24hr: g, or UPC ratio (g/g))	Post treatment Cr	Outcome
Abro, Paras A, 2024[1]	63	Male	Sq cell ca. of skin	Cemiplimab	NA	13 mg/dL	513 mg/g	Fibrillary GN (DNAJB9+) + AIN	High dose glucocorticoids (no dose reported)	NA	NA	2.5 mg/dL	Renal recovery, negative UA
Alasadi eta, 2024[2]	64	Male	Mesothelioma	Nivolumab	NA	NA	Nephrotic range	MN (PLA2R +)	NA	Rituximab	Resolved	NA	Complete renal response (PLA2R -)
—	71	Female	Sq cell ca. of anal canal	Nivolumab	NA	NA	15 g	MCD	NA	Rituximab	Complete remission	NA	Tumor response with continued ICI
—	65	Male	Anaplastic thyroid carcinoma	Pembrolizumab	0.9 mg/dL	3.5 mg/dL	20 g	MCD	NA	Rituximab	0.5 g	0.9 mg/dL	Able to resume ICI
Athanasopoulo, D.[3], 2023	74	Female	RCC	Pembrolizumab	1.8 mg/dL	5.8 mg/dL	18 g	MCD	Prednisolone 1 mg/kg	Cyclosporine	Complete remission	Complete remission	Complete remission
Benes, B, 2022[4]	72	Male	Melanoma	Nivolumab	1.0 mg/dL	3.1 mg/dL	10 g	Immune complex GN	Prednisone	Rituximab	Resolved	Resolved	NA
Benyahia, R, 2023[5]	56	Male	NSCLC	Pembrolizumab	0.79 mg/dL	NA	15g/g	MN (PLA2R +)	Prednisone 1 mg/kg/day	Rituximab 375mg/m2/weekly x 1 month	Resolved	Complete remission	Complete renal recovery, PLA2R -, however tumor progression
—	67	Male	PUCa	Atezolizumab	0.88 mg/dL	1.3 mg/dL	4.6 g/g	MN (PLA2R +)	NA	Rituximab (1 g day 1 and day 15 then 500 mg reinfusion 4 months later)	Resolved	Resolved	Complete renal recovery, PLA2R -, Able to resume ICI
Bonilla, M. A, 2021[6]	80	Male	Metastatic Lung Adeno Ca.	Pembrolizumab	1.18 mg/dl	1.80 mg/dl	10.8 g/g	Immune complex GN	Prednisone 1 mg/kg	NA	0.2 g/g	1.19 mg/d baseline	Complete renal recovery
Bonilla, M, 2021[7]	88	Male	Sq cell ca of head and neck	Cemiplimab	1.3 mg/dl	2.2 mg/dl	11.9 g/g	MN (PLA2R + 149RU/ml)	Oral corticosteroids	NA	4.8 g/g	NA	Improved PLA2R + <2RU/ml, ICI not resumed, died of disease progression
Biswas, A. 2024[8]	77	Male	NSCLC	Pembrolizumab	1.0 mg/dL	1.1 mg/dL	6.38 g	Secondary amyloidosis	NA	NA	NA	NA	NA
Calder, M. B, 2023[9]	73	Male	HCC	Nivolumab	NA	Dialysis dependent AKI	Nephrotic range	IgA N	High dose glucocorticoids (no dose reported)	Rituximab	Resolved	Return to baseline	Dialysis discontinued
Chabannes, M, 2024[10]	65	Male	NSCLC	Pembrolizumab	1.22 mg/dL	6.45 mg/dL	3.4 g/g	IgA N	Oral corticosteroids 1 mg/kg	Cyclophosphamide	Improved	Improved	Cessation of hemodialysis, died of disease progression
Chemaly, E. R, 2020[11]	26	Female	Hodgkin lymphoma	Nivolumab	0.6 mg/dL	2.3 mg/dL	19.6 g	MCD	Methylprednisolone 40 mg IV daily, then dexamethasone 4 mg daily	NA	1.55 g	0.6 mg/dL	Renal recovery, died of disease progression
Chen, M. 2021[12]	74	Male	Metastatic Lung Adeno Ca.	Tislelizumab	0.67 mg/dL	0.84 mg/dL	20.2 g	MN (THSD7A +)	Methylprednisolone 60 mg/day -> prednisone 60 mg/day	rituximab	0.17 g	0.57 mg/dL	Complete renal response (THSD7A -)
Chewcharat, A, 2024[13]	69	Male	Metastatic thyroid Ca	Nivolumab/ ipilimumab	1.1 mg/dL	1.9 mg/dL	2 g/g	Fibrillary GN (DNAJB9 not reported)	Rituximab	NA	0.18 g/g	1.2 mg/dL	Renal recovery
Cruz-Whitley, J, 2020[14]	75	Female	Non-Hodgkin lymphoma	Nivolumab	1.07 mg/d	1.63 mg/d	12.7 g/g	MPGN	Prednisone 40 mg daily	NA	1.1 g/g	1.17 mg/dL	Renal recovery, died of disease progression
DiFranza, L. T, 2023[15]	59	Female	SCLC	Atezolizumab	1.2 mg/dL	5.56 mg/dL	1.31 g/g	Fibrillary GN (DNAJB9+)	Prednisone 60 mg/day	NA	0.41 g/g	1.95 mg/dL	Partial recovery
—	62	Male	SCLC	Atezolizumab	1.0 mg/dL	5.73 mg/dL	NA	Fibrillary GN (DNAJB9+)	NA	NA	NA	NA	Died before tx started
Dougherty, S. C, 2021[16]	70	Female	NSCLC	Ipilimumab	0.9 mg/dL	2.5 mg/dL	NA	IgA N	Prednisone 60 mg/day	NA	NA	No improvement	No improvement, hospice
Gakiopoulou, H,2019[17]	69	Female	Metastatic lung Ca.	Pembrolizumab	NA	1.6 mg/dL	16.2 g	Lupus like Membranous Glomerulopathy	NA	NA	NA	NA	NA

Gerardine, S, 2021[18]	44	Male	Lymphoepithelioma-like Ca of liver	Nivolumab	1.0 mg/dL	1.8 mg/dL	5.26 g/g	Lupus like nephritis (low C3 58 mg/dl, elevated ANA 1:1280 negative dsDNA and ANCA) with MPGN features	Pulse steroids and then prednisone 60 mg/day	Cyclophosphamide 75 mg/day	1.5 g/g	1.5 mg/dL	Partial recovery
Hanif, I. H. 2024[19]	80s	Male	Metastatic RCC	Pembrolizumab	2.0 mg/dL	6.0 mg/dL	4.5 g	Lupus like nephritis ("full- house" IM pattern)	Methylprednisolone 250 mg pulse daily for 3 days then prednisone 60 mg daily	Rituximab 1 g x 2 doses 2 weeks apart, initiated hemodialysis	NA	NA	Partial recovery, hemodialysis discontinued
Hoshina, A. 2023[20]	65	Male	Lung Adeno Ca.	Pembrolizumab	0.59 mg/dL	3.0 mg/dL	2.22 g/g	Crescentic GN due to anti- GBM with Membranous Nephropathy and ATIN	Methylprednisolone 500 mg daily for 3 days, then prednisolone 1 mg/kg/day	Prednisolone tapered to 2.5 mg/day	0.45 g/g	1.54 mg/dL	Partial recovery
Kalaria, A. 2023[21]	76	Male	Malignant Mesothelioma	Nivolumab + Ipilimumab	0.8 mg/dL	2.4 mg/dL	0.42 g/g	MN (PLA2R +)	Rituximab 1g x 2 doses 14 days apart	Prednisone taper	0.26 g/g	1.62 mg/dL	Recovery
Kethineni, R, 2022[22]	72	Male	Testicular Ca	Nivolumab + Ipilimumab	1.2 mg/dL	2.56 mg/dL	9.9 g/g	MCD	Prednisone at 1 mg/kg	NA	NA	1.5-1.6 mg/dL (new baseline)	Partial recovery
Kim, D. W. 2021[23]	46	Female	Malignant melanoma	Pembrolizumab	0.66 mg/dL	NA	3.2 g/g	FSGS	None	Irbesartan + Furosemide (Pembrolizumab stopped)	0.20 g/g	NA	Complete recovery
Kim, Y. 2021[24]	55	Male	NSCLC	Durvalumab	0.98 mg/dL	NA	11.2 g	MN (PLA2R +)	None	ACEi, diuretics, no immunosuppressive therapy given	Improved, level not reported	Stable at 1.2 mg/dL	Improved
Kitchlu, A. 2016[25]	43	Male	Hodgkin lymphoma	Pembrolizumab	NA	3.93 mg/dL	10.3 g	MCD	Prednisone taper starting with 2mg/kg/day	NA	3.1 g	1.50 mg/dL	Improved, died of disease progression
	45	Male	Metastatic melanoma	Ipilimumab	0.80 mg/dL	NA	9.5 g	MCD	Prednisone at 1 mg/kg	NA	Remission	Stable 0.72 mg/dL	Proteinuria resolved, died of disease progression
Lapman, S, 2020[26]	42	Male	Colorectal Ca.	Pembrolizumab	1.0 mg/dL	1.58 mg/dL	7.6 g	Secondary AA amyloidosis	NA	Tocilizumab, hemodialysis	NA	NA	No renal recovery despite cancer remission
_	75	Male	Metastatic SCLC	Nivolumab	0.76 mg/dL	NA	9.4 g/g	Secondary AA amyloidosis	Prednisone	Colchicine	5.1 g/g	Stable	Stable renal function, proteinuria improved
_	81	Male	Metastatic melanoma	Nivolumab	1.0 mg/dL	7.7 mg/dL	4.6 g	Secondary AA amyloidosis	Prednisone	Colchicine, hemodialysis	No improvement	No improvement	No improvement
Leuprecht, L, 2020[27]	62	Male	Metastatic sarcoma	Nivolumab + Ipilimumab	0.80 mg/dL	4.5 mg/dL	5.1 g	IgA N	Pulse steroids	Mycophenolate	NA	2.7 mg/dL	Improved however mycophenolate added due to steroid related side effects
Lin, J. S. 2020[28]	60	Male	Malignant pleural mesothelioma	Nivolumab	0.82 mg/dL	1.25 mg/dL	13.4 g	MN (PLA2R+)	NA	Rituximab 1g x 2 doses 14 days apart	2.9 g	1.01 mg/dL	Renal recovery, PLA2R -, continued to receive nivolumab
Mamlouk, O, 2020[29]	71	Male	Malignant melanoma	Nivolumab + Ipilimumab	1.4 mg/dL	2.21 mg/dL	7.7 g/g	IgA N, superimposed MCD	Prednisone at 1 mg/kg	Rituximab, subsequently added Acthar gel 80mg twice weekly due to nephrotic syndrome not suppressed and intolerant to steroids	0.2 g/g	1.5mg/dL	Complete renal recovery, melanoma went into remission
Mansoor, S, 2020[30]	55	Male	Bladder Ca.	Pembrolizumab	1.0 mg/dL	1.4 mg/dL	19.5 g	FSGS	Steroids given, no dose available	NA	Improved	Improved	Renal improvement
Mitarai, Y. 2022[31]	70	Male	SCLC	Pembrolizumab	NA	NA	3.0 g/g	IgA N	Methylprednisolone	NA	0.5 g/g	No recovery	No renal recovery
Ngo, D. 2023[32]	67	Male	Mesothelioma	Nivolumab + Ipilimumab	0.79 mg/dL	3.78 mg/dL	3.7 g/g	FSGS (NOS) + AIN	Oral steroids	NA	No improvement	No improvement	No improvement
Nikmar A. 2023[33]	71	Male	Lung Adeno Ca.	Pembrolizumab	0.80 mg/dL	NA	4.2 g/g	MN	No medical treatment	Stopped Pembrolizumab	1.2 g/g in 2 months then 0.8 g/g	NA	Improved proteinuria
Okamoto, M, 2020[34]	61	Male	Gastric Ca	Nivolumab	NA	NA	5.9 g/g	Secondary MN (PLA2R -)	Prednisolone 40 mg/day	NA	NA	NA	Complete remission
Oki, R. 2020[35]	75	Male	NSCLC	Pembrolizumab	0.65 mg/dL	0.79 mg/dL	1.0 g/g	IgA N + tubulointerstitial nephritis	No medical treatment	Stopped Pembrolizumab	0.7 g/g after 6 months off pembrolizumab	Remained stable	Renal recovery, NSCLC relapse required resuming chemo
Oleas, D. 2023[36]	72	Male	Lung Adeno Ca.	Atezolizumab	1.1 mg/dL	NA	6.3 g	MN (PLA2R+)	No steroids given	ACEi, Rituximab	3.5g (6 months after rituximab), new dose given	NA	Negativization of PLA2R achieved 7 months after rituximab

Shim, J. 2021[37]	68	Male	Gastric Ca.	Nivolumab	0.77 mg/dL	1.96 mg/dL	1.73 g/g	Glomerular endothelial injury	No medical treatment	Stopped nivolumab	Resolved	Renal recovery	Able to resume nivolumab
Irifuku T. 2020[38]	59	Male	RCC	Nivolumab	1.13 mg/dL	3.09 mg/dL	1.5 g	Immune-mediated glomerulonephropathy + acute tubulointerstitial nephritis	Methylprednisolone 1 g/day for 3 days, then prednisolone 40 mg/day.	NA	0.17 g/g	1.32 mg/dL	Did not need to hold nivolumab
Shringi, S. 2024[39]	73	Male	Sq cell Ca. or lung	Pembrolizumab	NA	9.4 mg/dL	N A	IgA N	Pulse steroids	NA	No improvement	No improvement	Transitioned to hospice
Tanabe, K. 2020[40]	78	Male	Gastric Ca.	Nivolumab	0.72 mg/dL	1.45 mg/dL	3.59 g/g	IgA N	Prednisolone 0.6mg/kg/day	NA	0.18 g/g	1.2 mg/dL	Renal improvement
Vaughan, E. 2020[41]	57	Male	Metastatic Sq cell Ca. of tongue	Nivolumab	0.79 mg/dL	2.28 mg/dL	18.9 g	MCD	Prednisolone 75 mg/day	Furosemide 80 mg	No improvement	No improvement	No improvement, transition to palliative management
Wang R. 2020[42]	72	Male	Epitheloid mesothelioma of left lung	Pembrolizumab	NA	1.66 mg/dL	N A	IgA N	No medical treatment	Pembrolizumab Stopped for 2 months	NA	Partial recovery	Partial renal recovery
Yang, Z. 2023[43]	68	Male	NSCLC	Pembrolizumab	0.52 mg/dL	6.13 mg/dL	9.48 g/g	C3GN	Prednisone 60 mg/day	Cyclophosphamide, hemodialysis	3.19 g/g	4.12 mg/dL	Died 2 weeks after discontinuing hemodialysis
Yildiz, G. 2024[44]	7	Male	Malignant melanoma	Nivolumab	NA	NA	4.1 g	IgA N	Prednisolone 2 mg/kg/d	NA	Complete recovery	NA	Complete recovery in 6 months

1. Abro PA, Abro SG, Fatima H, et al. Rare Case of Fibrillary Glomerulonephritis with Concomitant Acute Interstitial Nephritis Related to Cemiplimab Use. *Journal of the American Society of Nephrology* 2024;35(10) doi: 10.1681/ASN.20249q06kxxa
2. Alasadi Y, Arani N, Tchakarov A, et al. Rituximab in Treatment of Glomerulonephritis Induced after Immune Checkpoint Inhibitors. *Journal of the American Society of Nephrology* 2024;35(10) doi: 10.1681/ASN.20248tqmmpq3
3. Athanasopoulou D, Lionaki S, Skalioti C, et al. Drug-Induced Podocytopathies: Report of Four Cases and Review of the Literature. *Life-Basel* 2023;13(6) doi: ARTN 1264

10.3390/life13061264

4. Benes B, Muir KL, Foster KW, et al. Immunotherapy-Induced Glomerulonephritis: Whodunit? *Journal of the American Society of Nephrology* 2022;33(11):381-81.
5. Benyahia R, Lazareth H, Flahault A, et al. Membranous Nephropathy After Exposure to Immune Checkpoint Inhibitors. *Kidney International Reports* 2023;8(9):1892-98. doi: 10.1016/j.ekir.2023.06.001
6. Bonilla MA, Bijol V, Corona A, et al. A Case of Immune-Complex-Mediated Glomerulonephritis Associated with Pembrolizumab. *Journal of the American Society of Nephrology* 2021;32(10):832-32.
7. Bonilla MM, B.; Khan, N.; Bijol, V.; Jhaveri, K. D.; Wanchoo, R. Phospholipase A2 receptor antibody mediated membranous nephropathy associated with cemiplimab. *Journal of Onco-Nephrology* 2021
8. Biswas A, Aggarwal N, Goswamy V. Secondary amyloidosis in a cancer patient on pembrolizumab-an unusual finding. *Nephrol Dial Transpl* 2024;39:I660-I62.
9. Calder MB, Wiegley N, Jen KY, et al. IgA Glomerulonephritis in the Setting of Immune Checkpoint Inhibitor Use Successfully Treated with Rituximab. *Journal of the American Society of Nephrology* 2023;34(11):483-83.

10. Chabannes M, Lisri Z, Lang S, et al. Immune checkpoint inhibitor therapy associated with IgA nephropathy: a case report and literature review. *Front Immunol* 2024;15 doi: ARTN 1393901

10.3389/fimmu.2024.1393901
11. Chemaly ERSS, J.; Hussain, F.; Mogollon-Duffo, F.; Laber, D. A.; Durr, J. A.; Khalighi, M. A.; Lopez del Valle, R. Minimal change disease and acute interstitial nephritis in a patient with Hodgkin's lymphoma treated with nivolumab. *Journal of Onco-Nephrology* 2020:81-86.
12. Chen MJ, Zhang L, Zhong W, et al. Case Report: THSD7A-Positive Membranous Nephropathy Caused by Tislelizumab in a Lung Cancer Patient. *Front Immunol* 2021;12 doi: ARTN 619147

10.3389/fimmu.2021.619147
13. Chewcharat A, Chowdhury RBZ, Petrosyan R, et al. Fibrillary Glomerulonephritis in a Patient on Dual Immune Checkpoint Inhibitor and Tyrosine Kinase Inhibitor Therapy. *Journal of the American Society of Nephrology* 2024;35(10) doi: 10.1681/ASN.2024vzs68qf7
14. Cruz-Whitley JG, N.; Jen, K. Y.; Young, B. Membranoproliferative Glomerulonephritis Associated with Nivolumab Therapy. *Case Reports in Nephrology* 2020
15. DiFranza LT, Chafouleas E, Katipally S, et al. Crescentic Fibrillary Glomerulonephritis in the Setting of Immune Checkpoint Inhibitor Therapy: A Report of Two Cases. *Glomerular Dis* 2023;3(1):69-74. doi: 10.1159/000528881 [published Online First: 20221230]
16. Dougherty SC, Desai N, Cathro HP, et al. IgA Nephropathy Secondary to Ipilimumab Use. *Case Rep Nephrol Dial* 2021;11(3):327-33. doi: 10.1159/000519169 [published Online First: 20211115]
17. Gakiopoulou H, Korkolopoulou P, Havaki S, et al. Lupus-like membranous glomerulopathy in a patient under pembrolizumab: report of a rare case. *Virchows Arch* 2019;475:S372-S73.
18. Gerardine S, Seshan S, Latcha S. Lupus Like Immune Complex Gn in a Cancer Patient on Pd1-Inhibitor Therapy. *Am J Kidney Dis* 2021;77(4):596-96.
19. Hanif IH, Abdelrahim S, Al Shaarani M, et al. Immune Checkpoint Therapy-Induced Lupus Nephritis. *Kidney Int Rep* 2024;9(5):1534-37. doi: 10.1016/j.ekir.2024.02.1396 [published Online First: 20240216]
20. Hoshina A, Endo S. Anti-glomerular basement membrane glomerulonephritis concurrent with membranous nephropathy and acute tubular interstitial nephritis in a lung cancer patient treated with pembrolizumab. *Cen Case Rep* 2023;12(2):230-36. doi: 10.1007/s13730-022-00750-x
21. Ahmad S, Kaldas H, Sethi S. A Case of Immune Checkpoint Inhibitor Associated Pla2r Membranous Nephropathy and Concurrent Acute Interstitial Nephritis Treated with Anti-Cd20 Therapy. *Am J Kidney Dis* 2023;81(4):S92-S93.
22. Kethineni R, Ohri R, Barry M, et al. Minimal Change Disease With Immune Checkpoint Inhibitors. *Journal of the American Society of Nephrology* 2022;33(11):380-81.
23. Kim DW, Jeon H, Kim S, et al. Pembrolizumab-induced focal segmental glomerulosclerosis A case report. *Medicine* 2021;100(43) doi: ARTN e27546

10.1097/MD.0000000000027546
24. Kim Y. Relapse of membranous nephropathy with cancer immunotherapy. *Clin Kidney J* 2021;14(1):418-20. doi: 10.1093/ckj/sfz074
25. Kitchlu A, Fingrut W, Avila-Casado C, et al. Nephrotic Syndrome With Cancer Immunotherapies: A Report of 2 Cases. *Am J Kidney Dis* 2017;70(4):581-85. doi: 10.1053/j.ajkd.2017.04.026
26. Lapman SW, W. L.; Parikh, R.; Khanin, Y.; Bijol, V.; Wanchoo, R.; Jhaveri, K. D. Immune checkpoint inhibitor-associated renal amyloid A amyloidosis. *Journal of Onco-Nephrology*
27. Iga nephropathy in the setting of dual immune checkpoint inhibitor use. Kidney week; 2020.
28. Lin JS, Wang DY, Mamlouk O, et al. Immune checkpoint inhibitor associated reactivation of primary membranous nephropathy responsive to rituximab. *J Immunother Cancer* 2020;8(2) doi: 10.1136/jitc-2020-001287 [published Online First: 2020/10/07]
29. Mamlouk OW, B. A case of relapsing immunoglobulin A nephropathy secondary to immunotherapy. *Journal of Onco-Nephrology* 2020

30. Case of FSGS in a patient on pembrolizumab. Kidney week; 2020.
31. Mitarai Y, Nakashima K, Fukunaga S, et al. IgA Nephropathy that Developed as an Immune-related Adverse Event of Pembrolizumab Complicated with Interstitial Nephritis. *Internal Med* 2022;61(13):2013-17. doi: 10.2169/internalmedicine.7115-2
32. Triple Whammy with Immune Checkpoint Inhibitor Therapy: A Case Report. Kidney international; 2023.
33. Nimkar A, Mehtabdin K, Wang XT, et al. Pembrolizumab-Associated Nerve Epidermal Growth Factor-Like 1 Membranous Nephropathy. *Kidney International Reports* 2023;8(3):676-79. doi: 10.1016/j.ekir.2022.12.018
34. A case of membranous nephropathy after nivolumab administration. American society of nephrology; 2020.
35. Oki R, Hirakawa Y, Kimura H, et al. Renal Effects after Pembrolizumab Treatment for Non-small Cell Lung Carcinoma. *Internal Med* 2020;59(7):977-81. doi: 10.2169/internalmedicine.3928-19
36. Oleas DS, A. Case report: Anti-PLA2R positive membranous nephropathy associated with atezolizumab. *Journal of Onco-Nephrology* 2023
37. Shim J, Tochio A, Ueda N, et al. Nivolumab-associated glomerular endothelial injury in a patient with gastric cancer. *Cen Case Rep* 2021;10(4):576-81. doi: 10.1007/s13730-021-00610-0
38. Irifuku T, Satoh A, Tani H, et al. Acute tubulointerstitial nephritis and IgM deposits on glomerular capillary walls after immunotherapy with nivolumab for metastatic renal cell carcinoma. *Cen Case Rep* 2020;9(1):48-54. doi: 10.1007/s13730-019-00424-1
39. Shringi S, Gohh RY. The Final Hit: A Case of IgA Nephropathy with Pembrolizumab. *Journal of the American Society of Nephrology* 2024;35(10) doi: 10.1681/ASN.2024mng5x93n
40. Tanabe K, Kanzaki H, Wada T, et al. Nivolumab-induced IgA nephropathy in a patient with advanced gastric cancer A case report. *Medicine* 2020;99(21) doi: ARTN e20464

10.1097/MD.0000000000020464
41. Vaughan E, Connolly E, Hui M, et al. Minimal change disease in a patient receiving checkpoint inhibition: Another possible manifestation of kidney autoimmunity? *Cancer Rep-Us* 2020;3(5) doi: ARTN e1250

10.1002/cnr2.1250
42. Wang R, Das T, Takou A. IgA nephropathy after pembrolizumab therapy for mesothelioma. *Bmj Case Rep* 2020;13(11) doi: ARTN e237008

10.1136/bcr-2020-237008
43. Yang Z, Xu H, Gou SJ, et al. Pembrolizumab induced-C3 glomerulonephritis and RBC cast nephropathy: a case report. *Bmc Nephrol* 2023;24(1) doi: ARTN 145

10.1186/s12882-023-03202-5
44. Yildiz G, Bayram MT, Ünlü SM, et al. Nivolumab-associated IgA Nephropathy in a Child With Malignant Melanoma. *J Pediat Hematol Onc* 2024;46(7):e534-e36. doi: 10.1097/Mph.0000000000002931
